# The European intercomparison of in-vivo monitoring laboratories: the EIVIC-2020 project

**DOI:** 10.1007/s00411-024-01060-9

**Published:** 2024-02-27

**Authors:** D. Franck, O. Meisenberg, T. Beaumont, W. Buchholz, M. A. López, J. F. Navarro, B. Pérez, K. Hürkamp, B. Breustedt, F. Vanhavere

**Affiliations:** 1 Institut de Radioprotection et de Sûreté Nucléaire (IRSN), 92260 Fontenay-aux-Roses, France; 2 Federal Office for Radiation Protection (BfS), 85764 Oberschleißheim, Germany; 3 Centro de Investigaciones Energéticas, Medioambientales y Tecnológicas (CIEMAT), 28040 Madrid, Spain; 4 European Radiation Dosimetry Group e. V. (EURADOS), 85764 Oberschleißheim, Germany; 5 KIT—Karlsruhe Institute of Technology, 76344 Eggenstein-Leopoldshafen, Germany; 6 Belgian Nuclear Research Centre (SCKCEN), 2400 Mol, Belgium

**Keywords:** Whole-body counting, In-vivo monitoring, Intercomparison, Phantom, Gamma emitters

## Abstract

The EIVIC project was launched in 2020, and the main goal was the organisation of a European intercomparison of in-vivo monitoring laboratories dealing with direct measurements of gamma-emitting radionuclides incorporated into the body of exposed workers. This project was organised jointly by members of EURADOS Working Group 7 on internal dosimetry (WG7), the Federal Office for Radiation Protection (BfS, Germany) and the Radioprotection and Nuclear Safety Institute (IRSN, France). The objective was to assess the implementation of individual-monitoring requirements in EU Member States on the basis of in-vivo measurements and to gain insight into the performance of in-vivo measurements using whole-body counters. In this context, a total of 41 in-vivo monitoring laboratories from 21 countries, together with JRC (EC) and IAEA participated. The results were submitted in terms of activity (Bq) of the radionuclides identified inside phantoms that were circulated to all participants. The measured data were compared with reference activity values to evaluate the corresponding bias according to the standards ISO 28218 and ISO 13528. In general, the results of the different exercises are good, and most facilities are in conformity with the criteria for the bias and z-scores in the ISO standards. Furthermore, information about technical and organisational characteristics of the participating laboratories was collected to test if they had a significant influence on the reported results.

## Introduction

A monitoring programme for occupational intake of radionuclides is one part of the general radiation protection programme, which is requested by the EC directive 2013/59/EURATOM (EC 2014). In particular, for the individual monitoring of workers, the Directive requires systematic monitoring based on individual measurements performed by a dosimetry service. In cases where workers are liable to receive significant internal exposure, member states have to set up an adequate system for monitoring which comprises regular in-vivo measurements using whole-body counters.

In 2018, the European Commission published in its Radiation Protection Series the document Technical Recommendations for Monitoring Individuals for Occupational Intakes of Radionuclides as RP 188 (RP 2018). This guidance document emphasises that for quality assurance of the measurement results, it is essential that the laboratories performing whole-body counting regularly participate in suitable interlaboratory comparisons.

In this frame, the European Commission funded already in the late 1990s an interlaboratory comparison of whole-body counting on a European level (Thieme et al. 1998). In 2019, another call for tender (ENER/D3/2019–158) was launched with the objective to assess the implementation of the individual-monitoring requirements of the Basic Safety Standards (BSS) Directive in EU member states based on in-vivo measurements and to receive an overview of the performance of in-vivo measurements using whole-body counters (WBC).

In accordance with the tender specifications, the project “European In-vivo Intercomparison Exercise 2020” (EIVIC-2020) was initiated jointly by the European Radiation Dosimetry Group (EURADOS), the Federal Office for Radiation Protection (BfS, Germany) and the Radioprotection and Nuclear Safety Institute (IRSN, France).

The objective of this paper is to present the organisation of the European intercomparison and to give a summary of the results of the different exercises put in place. Furthermore, information about technical and organisational characteristics of the participating laboratories was collected to test if they had a significant attribution with the quality of the reported results.

## Contacts with European laboratories performing whole-body counting

In previous efforts, members of the EURADOS Working Group “Internal Dosimetry” (WG7) collected the interest of in-vivo counting laboratories in a WBC European Intercomparison. National stakeholders who participated in former projects conducted by members of the EIVIC team were also contacted. These stakeholders were asked to act as national contact points by an official invitation letter, to disseminate information about this project and to nominate participants from their country.

**Fig. 1:**
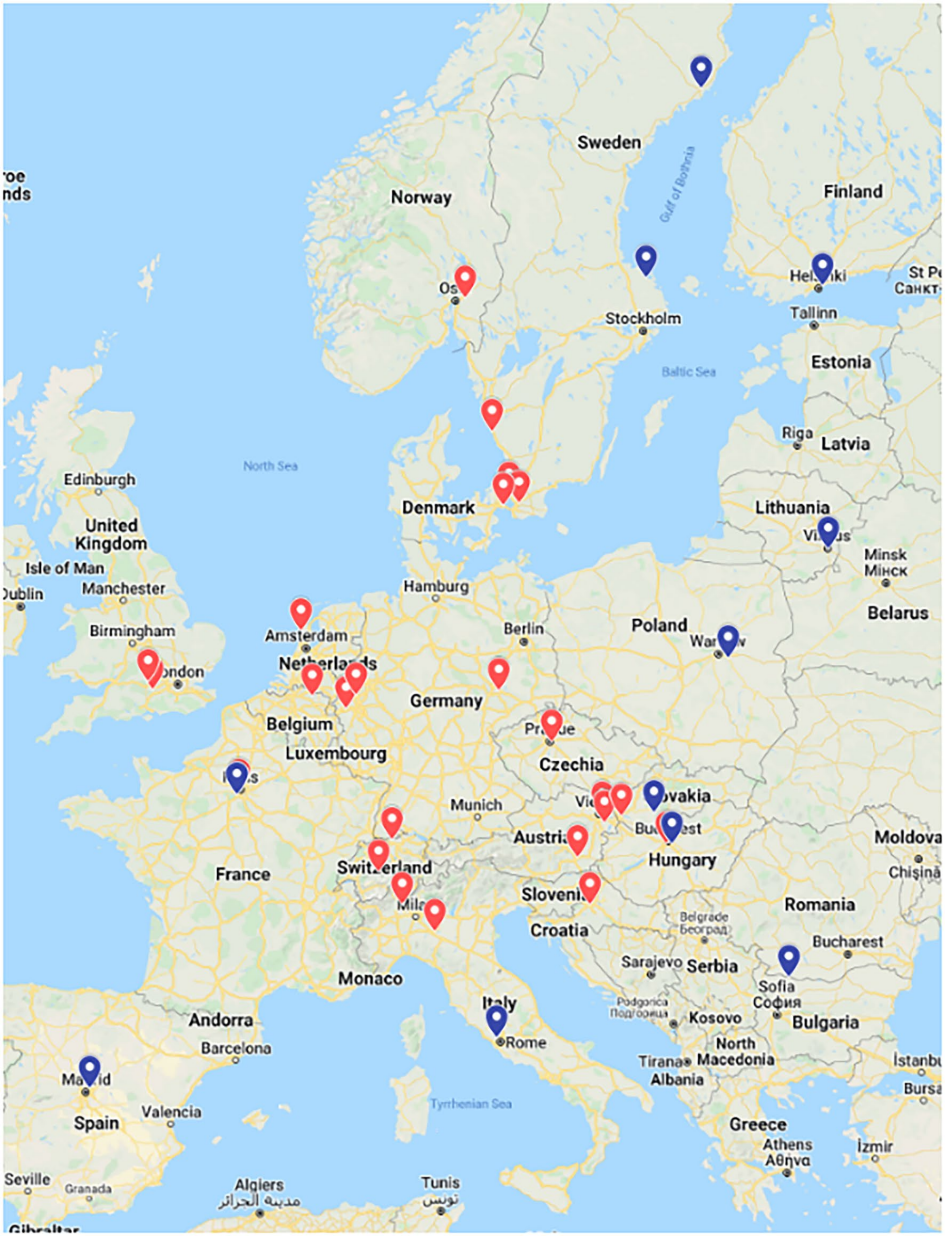
Distribution of participating laboratories, in red circuit organised by attended transport, in blue circuit organised by shipment (Map data: ^©^ 2022 Google, GeoBasis-DE/BKG, Inst. Geogr. Nacional.)

Based on these contacts, the final list of participants was established. Finally, 35 in-vivo monitoring laboratories from 21 countries, together with the Joint Research Centre (JRC) of the European Commission (EC) and the International Atomic Energy Agency (IAEA) participated in the EIVIC exercise. As seen in Fig. 1, at least one laboratory from all EU member states operating in-vivo counting facilities participated in the intercomparison (except for Romania for technical reasons). The laboratories were operated by research facilities, nuclear power plants, military installations, hospitals, commercial enterprises and national and regional agencies. Several laboratories conducted measurements with more than one whole-body counter and two laboratories did not report results after conducting the measurements, so that 41 results were finally received.

A questionnaire was sent to the participants to collect information about their equipment and their capabilities. This information was used to create a database that indicates the current situation of in-vivo monitoring in Europe and to be used for the statistical analysis of the results.

## Setup of the measurement campaign

The measurement campaign was started in the first week of May 2021 and finished at the end of November 2021.

The transport of the phantom to the participating facilities was conducted either as (i) a transport attended by a representative of the EIVIC team, or (ii) by shipment by a delivery company.

The principal points for the decision between attended transport and shipment were the distance, prospective journey time, the accessibility, temporal availability and subsequent placement in one of the tours as well as the possible requirement of customs clearance for facilities outside the EU. Finally, 24 facilities (with 26 WBC) took part in the attended tour, whereas 11 facilities (with 17 WBC) received the phantom by shipment.

In Fig. 1, the distribution of participating laboratories is indicated in red circuit organised by attended transport, and in blue circuit organised by shipment.

## Materials and methods

### Phantoms, sources, and measurement tasks

**Fig. 2:**
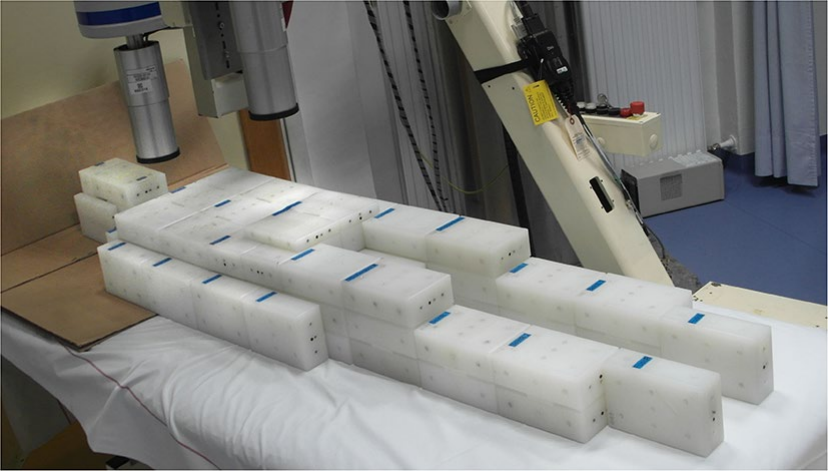
Photo of the phantom in the P4/70 kg configuration, erected in a whole-body counter with stretcher geometry

To simulate occupational internal exposures, anthropomorphic phantoms with different sizes (limited to phantoms resembling adults) and different radionuclides were chosen. The radionuclides were limited to radionuclides that are commonly encountered in occupational exposure situations or feature characteristics that are advantageous for the assessment of the proficiency of whole-body counters. For the EIVIC intercomparison, the type of phantom selected was the Saint Petersburg brick phantom (Kovtun et al. 2000). This phantom consists of rectangular bricks made from polyethylene, which can be set up in six shapes (P1–P6) resembling persons of weight 12–110 kg. The bricks contain holes, which can be filled with rod sources of known activities. It is considered as an appropriate method for calibration of whole-body counters (ICRU 2003). Two sizes of phantoms have been used for the EIVIC project: P4 and P5 corresponding to 70 kg (Fig. 2) and 90 kg persons, respectively.

Furthermore, the nuclides and the activities used in the exercise were also selected taking into account the availability of certified sources at the planned time of the exercise and activity limits defined by transport regulations.

For this intercomparison, four tasks were defined concerning measurement of phantoms equipped with radionuclide sources. For each phantom measurement task, one specific set of radionuclide sources was used. Each set contained a mixture of those radionuclides that were to be measured in the respective measurement task. The nuclides and phantoms used in the different tasks were:

- Task 1 called “Victor”: ^60^Co, ^133^Ba, ^137^Cs and ^40^K with phantom P4/70 kg
- Task 2 called “Emergency”: ^134^Cs, ^137^Cs and ^40^K with phantom P5/90 kg
- Task 3 called “Medicine”: ^68^Ge, ^88^Y and ^40^K with phantom P4/70 kg
- Task 4 called “Calibration”: ^133^Ba, ^152^Eu and ^40^K with phantoms P4 and P5.

Rod sources for Task 1 were taken from the stock of IRSN (Benyakoub 2017). Rod sources for Tasks 2, 3 and 4 were produced and qualified in the laboratories of BfS (Woidy and Meisenberg 2022). All sources were subject to the same quality-assurance (QA) measurements, performed at the whole-body counting laboratories at BfS, CIEMAT, KIT and IRSN before the start of the intercomparison exercise as described in Franck et al. (2023):

- The activity of the radionuclides in each single rod source for Tasks 2, 3 and 4 was determined using a precision balance (CP124S with draught shield, resolution 0.1 mg, Sartorius, Germany), which was subject to annual quality-assurance checks by an accredited service. Since the activity of the radionuclide solution was traceable, this yielded a traceable activity of each single source. However, the results were affected by the uncertainty of the weighing of small masses in the order of 10 mg.
- Each single rod source was measured with an HPGe gamma-spectroscopy detector (GMX series, n-type, Ortec, USA, calibrated with ^60^Co, ^133^Ba and ^137^Cs with traceability). Two geometries were used: a high-efficiency geometry where the source was placed in close contact horizontally on the detector; a low-efficiency geometry with approximately 13 cm between the horizontal source and the detector. For most radionuclides, measurements in both geometries were affected by true-coincidence summing (the low-efficiency geometry to a lesser extent but yet observable).
- All sets of sources were measured inside the phantoms that were assembled according to the respective measurement task. The measurements were conducted with the whole-body counters of each organiser of the project: with HPGe detectors at BfS, IRSN and KIT and with NaI(Tl) and broad-energy germanium (BEGe) detectors at CIEMAT.

Though the quality of the sources was validated by these different QA processes, discrepancies of several percents were observed between the results of the three processes. As a result, it was decided to use the robust means of the results reported by the participants as assigned values as explained in the next section.

For Task 3, given the short half-lives of the radionuclides used, two sets of sources were fabricated. The results were analysed for these two sets of sources separately (#1 and #2). The purpose of these measurements was mainly the detectability of the nuclides and the ability to measure their activities with good precision.

### Data evaluation

The laboratories had to identify and quantify the radionuclides present in the phantom. The activity and the uncertainty related to each result had to be expressed in Becquerel (Bq). The latter is given as the expanded uncertainty at 2σ indicating a coverage factor *k* equivalent to 2.

The laboratories reported results that were valid at the date of the measurement.

To compare all the results, decay correction to a reference date was conducted by the EIVIC-2020 team. For the measurement of Tasks 1, 2, 3 (set 1) and 4, 01/05/2021 was taken as the reference date, and for the Task 3 (set 2), it was 10/08/2021.

Decay correction performed by the EIVIC-2020 team based on an identical half-life for all laboratories and each radionuclide ensured that no additional source of possible errors and uncertainties was introduced in this step.

The data provided by the participating laboratories have been treated statistically using ProLab^™^ software. The statistical processing was the following:

1. Search for aberrant values by the Grubbs method (Stefansky 1972),
2. Relative bias: assessment of the laboratory performances according to standard ISO 28218 (ISO 2010),
3. Z-score: assessment of the laboratory performances according to standard ISO 13528 (ISO 2022).

Each participating in-vivo measurement facility was referred to using a code number. These codes are consistent throughout the paper, i.e., one code will always refer to the same facility. The allocation of numbers was made randomly by computer, and there is, therefore, no connection between the numbered codes and the names of the institutes.

The performance criteria relative bias and z-score are detailed below. The conformity of the results with these various criteria was used to qualify the proficiency of the laboratories.

### Assigned value

As explain before, the consensus value from participants was used, determined with a robust method according to ISO 13528 (ISO 2022). Robust mean refers to the arithmetic mean of the reported values without outliers and was calculated using the Q/Hampel method^1^. This method uses the Q method for the calculation of the robust standard deviation s* together with the Hampel estimator for the calculation of the robust location parameter x*. It is applied for the statistical analysis of interlaboratory studies. It was used to guarantee a homogeneous and robust analysis between the different tasks.

### Estimated bias

The relative bias is a measure of how close the assessed activity is to the target value (reference). According to ISO 28218 (ISO 2010), the relative bias must be within a range of -25% to + 50% relative to the target value. When the bias is outside the range of −25% to + 50%, the service laboratory shall make appropriate corrections in phantom calibration or measurement protocols to reduce or eliminate the bias.

The laboratory bias estimate is defined as a percentage. This performance test is calculated as follows:

$$\text{Bias}\,\left(\text{\%}\right)=\frac{x-X}{X}\times 100;$$

$x$: Result of the participating laboratory (Bq), $X$: Activity of the target value (assigned value) (Bq).

### Outliers (Grubbs Test)

Each data set was subjected to the Grubbs test to detect possible outliers at the ends of the distribution.

The test consists in calculating, for *n* values classified in ascending order of﻿ $x_{1}$, $x_{2}$, …, $x_{n}$, the test statistic ${G}_{p}$

$$\text{to test }x_{1}\text{:}\,\,G_{p} = \frac{{\overline{x} - x_{1}}}{s}\text{, to test }x_{n}\text{:}\,\,G_{p} = \frac{{x_{n} - \overline{x}}}{s},$$

with *S*: interlaboratory standard deviation, $x_{1}$: lowest population value, $x_{n}$ : highest population value, $\overline{x}$: mean of the *n* values of the population.

The value of ${G}_{p}$ is compared with a critical value that depends on the number *n* of values. If one of the extreme values is identified as an outlier, this value is discarded, and the test is repeated with the remaining set of values until no value is identified as an outlier anymore.

### Z-score estimation

The z-score is an indicator of the laboratory proficiency compared to that of the other laboratories, because it is correlated with the robust standard deviation. Thus, it depends directly on the dispersion of the results from the laboratories. The z-score is calculated by means of the following formula:

$$z=\frac{x-X}{\widehat{\sigma }}\times 100;$$

$x$: result of the participating laboratory (Bq), $X$: activity of the target value (Bq), $\widehat{\sigma}$: robust standard deviation for proficiency evaluation. According to the recommendations of ISO 13528 (ISO 2022), the current z-score criteria are

- $\left|{\text{z}}-{\text{score}}\right|$ ≤ 2: the result is satisfactory,
- 2 < $\left|{\text{z}}-{\text{score}}\right|<$ 3: the result is considered to give a warning signal,
- $\left|{\text{z}}-{\text{score}}\right|$ ≥ 3: the result is considered unacceptable (action signal).

It has to be noted that zeta-score was not used for the analysis. As described in ISO 13528, this estimator based on the uncertainties of measurement is only relevant for measurements done in the same condition for measurement and calibration. In the case of this intercomparison, because of the large diversity of the whole-body facilities in terms of detection system (NaI(Tl) or HPGe), type of calibration phantom of the labs and protocols used, it was decided not to use this estimator.

### Results of the different tasks

The number of the laboratories that participated in the different tasks and submitted results is given in Table 1. It can be observed that the number of reported results is not equal for all radionuclides and smaller for Task 3 (^68^Ge, ^88^Y and ^40^K probably because of measurement of not classical radionuclides) and for Task 4a and 4b (^133^Ba, ^152^Eu and ^40^K): 20/43 facilities (^152^Eu), probably because this task was dedicated to germanium detectors and not mandatory.

**Table 1:** Summary of results submitted by the laboratories in the EIVIC intercomparison for the different tasks (43 results were expected) (*)

Tasks	Number of participants	Number of results submitted
Task 1	43	
^60^Co		39
^133^Ba		39
^137^Cs		40
^40^K		28
Task 2	43	
^134^Cs		40
^137^Cs		40
^40^K		28
Task 3 (#1 and #2)	42	
^68^Ge		17
^88^Y		37
^40^K		29
Task 4a	25	
^133^Ba		21
^152^Eu		20
^40^K		17
Task 4b	33	
^133^Ba		31
^152^Eu		30
^40^K		21

(*) no data submitted by labs 26 and 40 after conducting the measurements

**Fig. 3:**
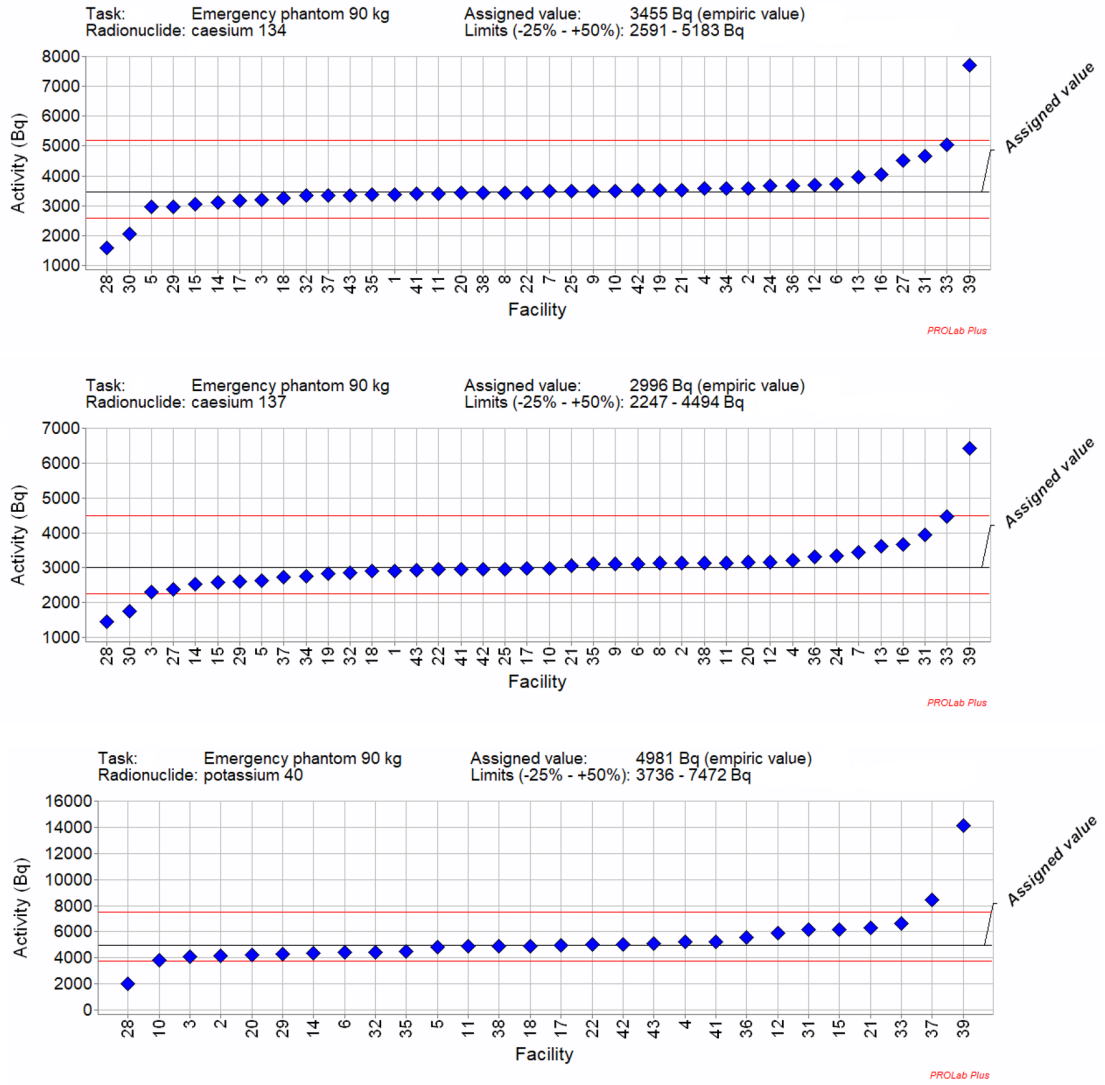
Representation of the reported activities of participants: ^134^Cs, ^137^Cs and ^40^K for Task 2 “Emergency”. Solid lines: in blue assigned activities (robust mean of the reported results); in red: + 50%/-25% criteria

**Fig. 4:**
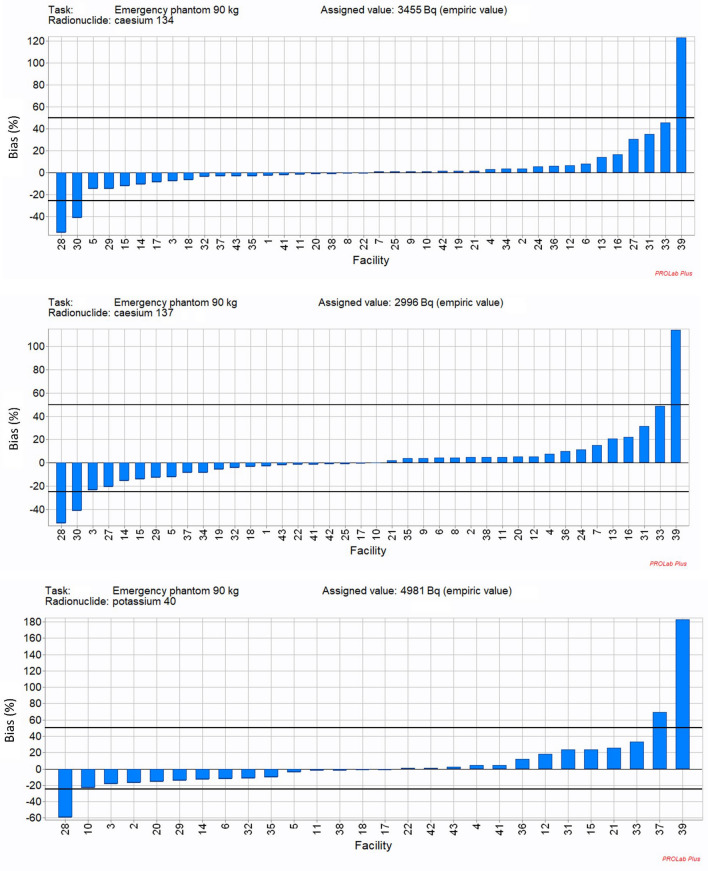
Representation of the bias (%) for the radionuclides of Task 2 “Emergency” (solid line: + 50%/-25% criteria)

**Fig. 5:**
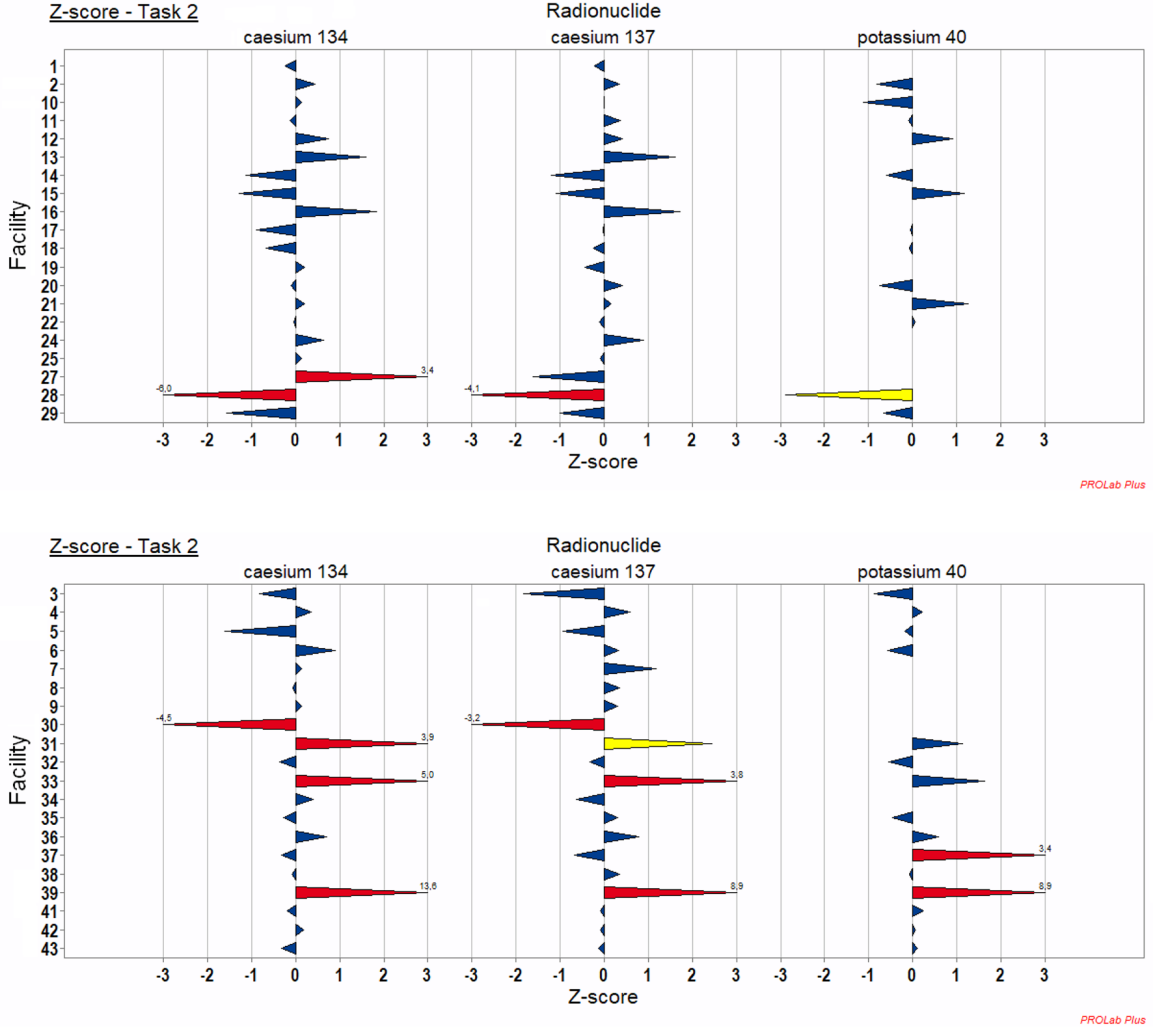
Representation of the z-score for the radionuclides of Task 2 “Emergency”. In blue |z-score|≤ 2: the result is satisfactory; in yellow 2 <|z-score|< 3: the result is considered to give a warning signal; in red |z-score|≥ 3: the result is considered unacceptable (action signal)

The reported activities, bias and z-score analysis are illustrated in Figs. 3, and for Task 2 “Emergency”. All results of the participant facilities for the other three tasks are summarised in Franck et al. (2023). It can be observed that the reported activities are quite good and distributed around the assigned value despite variabilities for some laboratories (Fig. 3). The bias and z-score analysis show that the results are also quite good according to the ISO standards (Fig. 4 and 5). It can be noted that for example lab 31 is in conformity for all radionuclides according to ISO 28218 but gives a warning signal for ^137^Cs, an action signal for ^134^Cs and is acceptable for ^40^K according to ISO 13528. These results can be explained by the difference of tolerance intervals and are detailed below.

The conclusion for this intercomparison can be summarised by the compliance reports for the participants of each task, with regard to the ISO 28218 (ISO 2010) and ISO 13528 (ISO 2022) standards. The results in compliance in function of these standard criteria are shown for Task 2 in Table 2, and for all the other tasks, they are given in the Appendices I to IV. Depending on the normative reference applied, it should be noted that a difference in conformity exists for several installations. Several reasons can explain this difference:

- The tolerance intervals are more restrictive according to ISO 13528 (ISO 2022) than to ISO 28218 (ISO 2010),
- The bias is a criterion which allows to assess the performance of an installation in relation to the “target” value (ISO 2010), and therefore independently of the other participants. The z-score is a performance estimator which depends on the dispersion of the results of participants (ISO 2022). It therefore allows to evaluate a facility compared to all the participating facilities (use of the robust standard deviation for capability evaluation).

**Table 2:** Compliance report for Task 2 “Emergency”

ISO standards	Task 2 “Emergency” (^134^Cs, ^137^Cs and ^40^K)
28218	Conform (all RN): 2, 3, 4, 5, 6, 10, 11, 12, 14, 15, 17, 18, 20, 21, 22, 29, 31, 32, 33, 35, 36, 38, 41, 42, 43 Conform (^134^Cs/^137^Cs only): 1, 7, 8, 9, 13, 16, 19, 24, 25, 27, 34, 37 Not conform (all RN): 28, 39 Not conform (^134^Cs/^137^Cs only): 30 Not conform (^40^K only): 37 No result: 23, 26, 40
13528	Acceptable (all RN): 2, 3, 4, 5, 6, 10, 11, 12, 14, 15, 17, 18, 20, 21, 22, 29, 32, 35, 36, 38, 41, 42, 43 Acceptable (^134^Cs/^137^Cs only): 1, 7, 8, 9, 13, 16, 19, 24, 25, 27, 34, 37 Acceptable (^137^Cs only): 27 Acceptable (^40^K only): 31, 33 Action signal (all RN): 39 Action signal (^134^Cs/^137^Cs only): 28, 30, 33 Action signal (^134^Cs only): 27, 31 Action signal (^40^K only): 37 Warning signal (^40^K only): 28 Warning signal (^137^Cs only): 31 No result: 23, 26, 40

Text in brackets denotes radionuclides (RN) to which the compliance statement is limited

According to the performance criteria, despite variabilities for some laboratories, the results for all tasks are good, both according to the bias criteria according to ISO 28218 and according to the z-score criteria according to ISO 13528. Task 3 (Medicine) presented the most difficulties to the participants to evaluate results, which is expressed by the fact that the number of participants who reported results for this task was small and almost exclusively limited to frequent participants in previous intercomparisons of IRSN (Berard and Franck 2011) or BfS. Nevertheless, it has been observed that the problems experienced by laboratories which submitted the most extreme results could generally be attributed to the calibration of their counters (in particular inappropriate adoption of lung calibrations for assessing activity in whole-body geometries).

However, the measurements were not carried out under equal conditions and with equal installations in all laboratories, particularly in terms of detection system (NaI(Tl) or germanium detectors of different sizes), calibration curves used (70 kg systematically or adapted to the configuration of the phantom), the duration of the measurement, the detector-phantom distances and the use of more or less realistic anthropomorphic phantoms for calibration. These results are therefore to be interpreted with care and must be considered as complementary elements allowing the laboratory to evaluate itself compared to other participants.

## Review of the main metrological and organisational characteristics of the facilities

As described in the first section, information about technical and organisational characteristics of the participating laboratories was collected. Several characteristics were used to test if they had a significant influence on the quality of the reported results. For this purpose, certain statistical tests were applied on the z-scores except those that were identified as outliers.

The tests were performed using the R software package, version 4.0.2 (R Core Team 2020). If not otherwise stated below, all reported z-scores (except outliers) from all four measurement tasks that involved phantoms (Task 1–4) were used for the tests. The following tests were conducted:

- Mann–Whitney *U* test to compare the central tendency of the values: This test predicates if data from the one subset are significantly greater or smaller than data from the other subset (alternate hypothesis) or not (null hypothesis). This test is comparable to a *t* test for normally distributed values. A two-sided test with correction for tied values was conducted.
- Siegel–Tukey test to compare the dispersion of the values: This test predicates if data from the one subset are significantly more or less dispersed than data from the other subset (alternate hypothesis) or not (null hypothesis). This test is comparable to an *F* test for normally distributed values. A two-sided test with correction for tied values and with adjustment of the medians was applied.

Reported results are the p-values, which are the maximum probabilities of obtaining the actual samples under the assumption of the samples originating from the same population. Small p-values indicate a significant difference between the sets of data, with the threshold set at 0.05 (i.e., confidence level of 95%). If the laboratories could be divided into more than two subsets (e.g., in the case of the measurement geometry, which was stretcher, inclined chair, chair, and standing), the first characteristic serves as the reference and data for all other characteristics are compared with the data for the reference characteristic. Additional to the p-values, box plots are presented: the box indicates the first quartile, median, and third quartile of the reported results, and the whiskers denote the minimum and maximum value.

## Type of participation in the intercomparison (visitation or shipment)

Twenty-four facilities (with 26 WBC) took part in the attended tour, whereas 11 facilities (with 17 WBC) received the phantom by shipment. The activities of the sources were as similar as possible in both phantoms for all tasks. Therefore, possible differences could have been caused in particular by the assistance during the setup of the phantoms in the attended tour, but also by the longer time that was available for the measurements in the shipment tour.

**Fig. 6:**
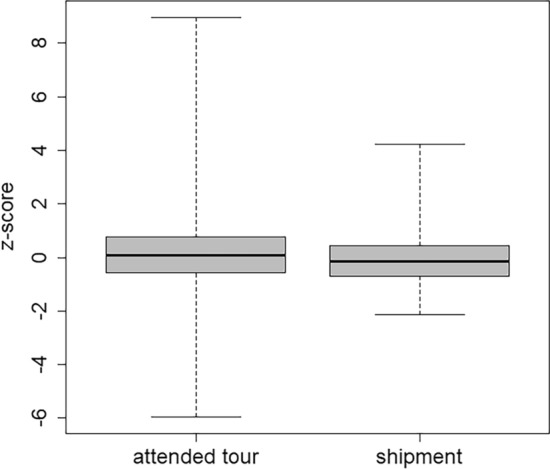
Box plot of the z-scores discriminated according to the type of participation

The difference between the central tendencies between attended tour and shipment (p-value 0.017; significantly different) is mostly caused by some very small z-scores (i.e., strong underestimation) in the attended tour (Fig. 6). This might be only a coincidence between those few labs that tended to strongly underestimate the results and the participation of these labs in the attended tour, but not a causal attribution between underestimation and attended tour. However, the higher number of reported results per laboratory in the shipment tour (12.6) as compared to the attended tour (10.5) can be explained. Not all laboratories conducted both measurements of Task 4 “Calibration” (with a phantom of 70 and with one of 90 kg), because of the limited time during the attended tour.

### Type of detector

Thirty whole-body counters conducted measurements with high-purity germanium (HPGe) detectors, and nine whole-body counters conducted measurements with sodium iodide (NaI(Tl)) detectors. NaI(Tl) detectors feature a reduced energy resolution compared to HPGe detectors, impeding the discrimination and identification of radionuclides with similar energy emissions in the sources. Two whole-body counters conducted the identification of the radionuclides with HPGe detectors and measured the activity of the identified radionuclides with additional NaI(Tl) detectors.

**Fig. 7:**
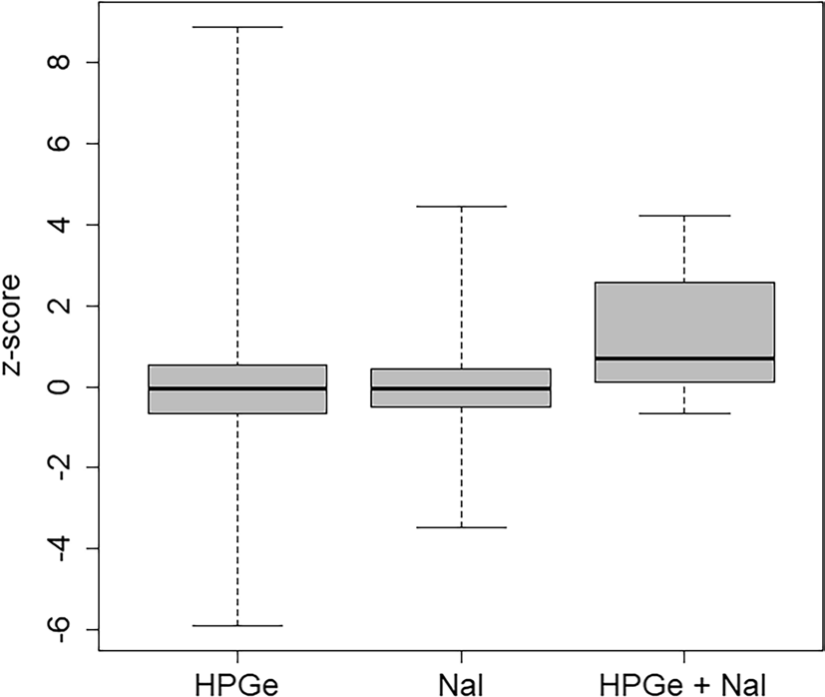
Box plot of the z-scores discriminated according to the type of detector

It can be seen that the performance of HPGe and of NaI(Tl) detectors is similar (Fig. 7; p-value for central tendency 0.39; p-value for dispersion 0.082, both not significantly different). The differing results for the combination of HPGe and NaI detectors are not significant because of the small number of results.

**Fig. 8:**
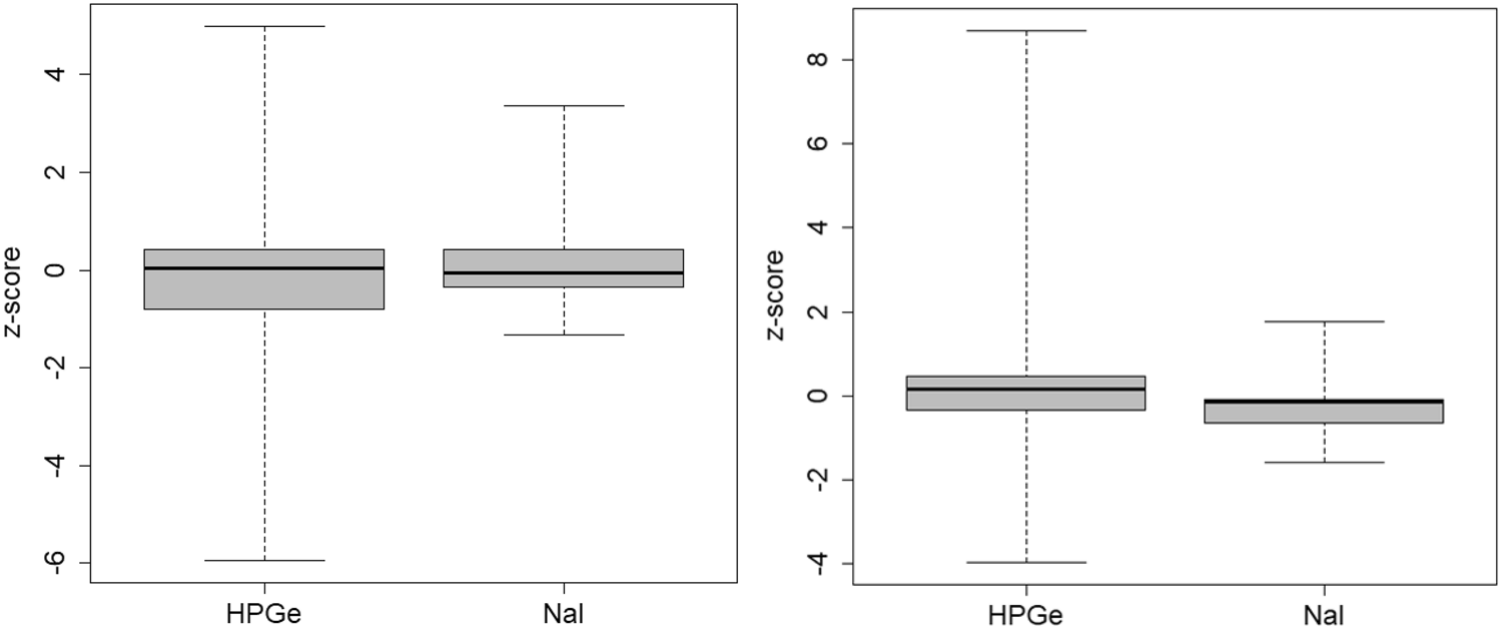
Box plot of the z-scores discriminated according to the type of detector for ^134^Cs (left) and ^137^Cs (right) in the Emergency task, in which both nuclides were present

It was also tested if different results between HPGe and NaI(Tl) detectors can be identified for ^134^Cs and ^137^Cs for the Emergency task (Task 2) in Fig. 8

The relevance of these nuclides in emergency response triggers the importance that they can be measured with NaI(Tl) detectors with good precision, which are often available for emergency measurements (e.g., as portable equipment or in mobile units of WBC). Each single radionuclide should not pose a difficulty for measurement by NaI(Tl) detectors, because of the small number of gamma emissions. However, in the Emergency task, both caesium isotopes were present together as they will usually be released during accidents in nuclear reactors so that the peak of ^137^Cs is overlapped by a peak of ^134^Cs in NaI(Tl) spectra. The activity of ^134^Cs can be calculated from undisturbed peaks, whereas this is not possible for ^137^Cs, since this nuclide features only one gamma emission.

It can be seen that ^137^Cs in the Emergency task was measured with NaI(Tl) detectors with a slight, insignificant underestimation as compared to HPGe detectors (p-value 0.26; for dispersion 0.84). It must be noted that the reference value was calculated as the robust mean of all reported results for this nuclide, so that the smaller ^137^Cs activities measured by NaI(Tl) detectors decreased the reference value. The underestimation could have been caused by the subtraction of the count rate of the combined ^134^Cs and ^137^Cs peak to calculate the activity of ^137^Cs only, which was necessary for NaI(Tl) detectors.

## Measurement of a 90 kg phantom with a 70 kg phantom calibration

A phantom with a body weight of 90 kg was used for the Emergency task. However, several laboratories usually calibrate their whole-body counters only with a phantom of 70 kg and apply that calibration for all people and phantoms to be measured regardless the body weights; others conduct calibrations with different phantoms of up to 70 kg. On the other hand, several laboratories apply calibrations also with phantoms of > 90 kg body weight. For the results of the Emergency task, the performance of laboratories with a calibration for up to 70 kg or 70 kg only and of those with a calibration also for 90 kg was compared.

**Fig. 9:**
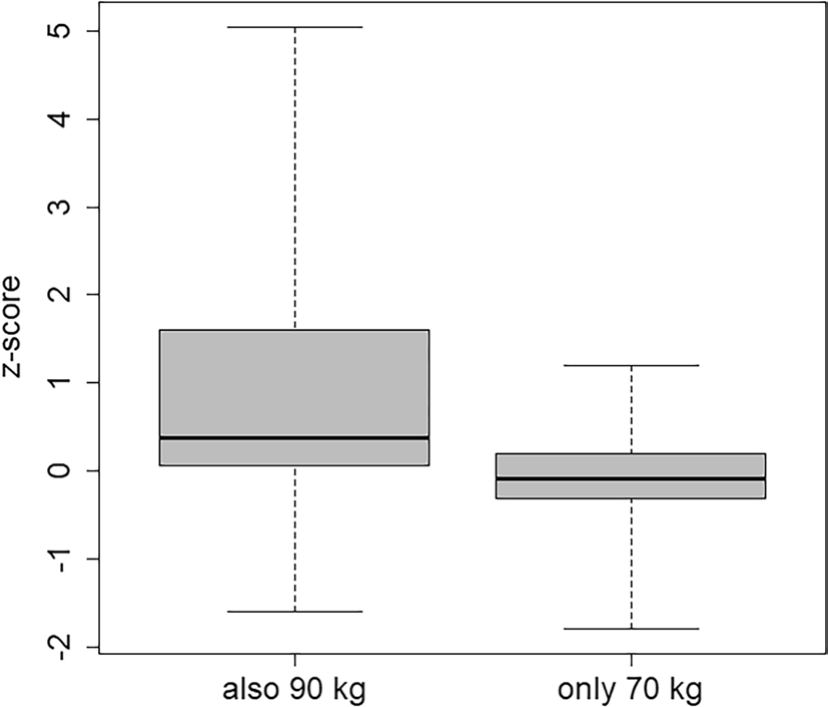
Box plot of the z-scores discriminated according to the phantom masses for calibration measurements

As shown in Fig. 9, the z-scores of those laboratories that calibrate their whole-body counters only at a mass of 70 kg are significantly smaller than the z-scores of those labs that applied a 90 kg calibration for the 90 kg measurement (p-value 2.5·10^–4^). It is sensible that smaller results were achieved if a 90 kg phantom was measured with a 70 kg calibration, because of the stronger attenuation of the emitted gamma radiation by the bigger phantom. It must be noted again that the reference value is the robust mean of all results.

## Measurement geometry

**Fig. 10:**
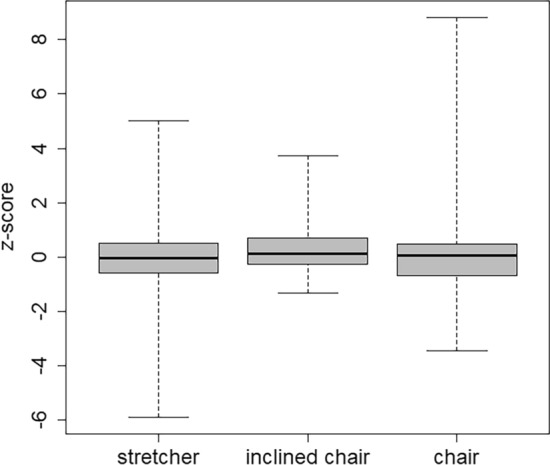
Box plot of the z-scores discriminated according to the type of geometry

The comparison of z-scores for different types of geometries is shown in Fig. 10. The whole-body counters that participated in the intercomparison conducted measurements in different geometries: stretcher/lying, chair/sitting, inclined chair, and standing. Because of the lack of statistical power, the only laboratory with standing geometry was excluded from the statistical analysis of the association between geometry and results. Central tendencies and dispersion of the results were not significantly different.

## Type of calibration phantom

The participating laboratories used different types of phantoms for the calibration of their whole-body counters (ICRU 1992, 2003): brick phantom (equal or similar to the one that was used in this intercomparison, 12 labs), bottle mannequin absorber phantom BOMAB (5 labs), other types of bottle phantoms (9 labs), Canberra Transfer Phantom (4 labs), computational phantoms for Monte-Carlo simulation (2 labs), the Lawrence–Livermore Lung Phantom (LLNL, 2 labs) and self-made phantoms (4 labs). To increase the statistical power of the comparison between bottle and brick phantoms, all types of bottle phantoms were summarised. Because of the small number of participating laboratories using LLNL lung phantoms and computational phantoms, these were excluded from the statistical analysis. Laboratories that conducted their calibrations with LLNL lung phantoms reported results that deviated from the reference value resulting in non-conformity as it was mentioned above, whereas laboratories conducting computational calibrations yielded acceptable results regarding their biases and z-scores.

**Fig. 11:**
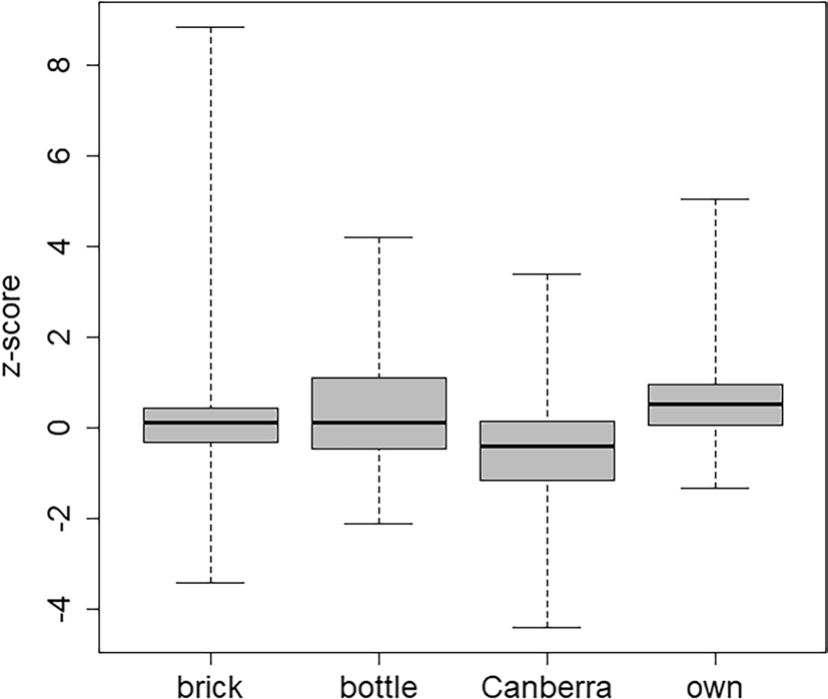
Box plot of the z-scores discriminated according to the type of calibration phantom

It can be seen in Fig. 11 that bottle phantoms and brick phantoms showed similar results regarding the central tendency (p-value 0.59), but different results regarding the dispersion (p-value 0.00093). The difference in the dispersion was influenced by some rather big under- and overestimations from laboratories with brick phantoms. With the Canberra phantom, results tended to be underestimated (p-value 0.00035) and with own phantoms results tended to be overestimated (p-value 0.0063; yet with small dispersion despite the different makeups of these phantoms).

## Conclusions

The objective of the EIVIC-2020 project was to assess the implementation of the individual-monitoring requirements in EU Member States based on in-vivo measurements and receive an overview of the capabilities and performance of whole-body counters in Europe. It was organised between 2020 and 2021 and dedicated to whole-body measurement of gamma emitters in several tasks selected that cover the range of possible measurements associated with different intake scenarios. In total, 43 installations from 21 countries took part in the proposed measurements. In a final project workshop at CIEMAT, Spain in June 2022, the EIVIC results were presented to participants, sharing with them experiences in the performance of the exercise and getting their feedback for future improvements.

Although the intercomparison is representative for the variability of the materials and methods used, and despite that the measurements are not carried out under the same conditions, the results are good according to the performance criteria with slight variabilities for some laboratories.

For the laboratories 28 and 39 with the most extreme biases over all tasks, following a discussion of the results with the laboratory staff, it turned out that they applied a calibration for lung measurements and tried to adapt the results to the whole-body geometry of the intercomparison. Non-conforming results are therefore to be interpreted with care and must be considered as complementary elements allowing the laboratory to evaluate itself compared to other participants.

A further analysis was therefore carried out to test if they had a significant attribution with the quality of the reported results with similar methods and measurement systems. Surprisingly, the results are quite similar for most of the participating laboratories, except for the dependency on phantom-size. This shows that size-dependent calibration factors should be used for the types of less common calibration phantoms. The quality of the results was rather independent from the metrological and organisational characteristics. The dispersion of the results within each investigated property was stronger than the difference between different properties. Therefore, attributable differences of these properties are small (no matter if significant or not).

Finally, the EIVIC project allowed knowing the current status of whole-body counters in Europe, to confirm their correct performance and suitable capabilities for in-vivo monitoring of gamma-emitting radionuclides incorporated in the human body.

## Data Availability

The authors declare that the data supporting the findings of this study are available within the paper. Further data are available in Franck et al. (2023).

## Appendix

See Tables 3, 4,5, 6.

**Table 3:** Compliance report for Task 1 “Victor”: ^60^Co, ^133^Ba, ^137^Cs and ^40^K

ISO standards	Task 1 “Victor” (^60^Co, ^133^Ba, ^137^Cs and ^40^K)
28218	Conform (all RN): 2, 3, 4, 5, 6, 10, 11, 12, 15, 17, 18, 20, 21, 22, 29, 32, 33, 35, 36, 38, 41, 42, 43 Conform (^60^Co, ^133^Ba and ^137^Cs only): 1, 7, 9, 13, 14, 19, 23, 24, 25, 34, 37 Conform (^60^Co, ^137^Cs and ^40^K): 31 Conform (^60^Co and ^137^Cs only): 8 Conform (^133^Ba only): 30 Conform (^137^Cs only): 27 Conform (^40^K only): 39 Not conform (all RN): 28 Not conform (^60^Co, ^133^Ba and ^137^Cs): 39 Not conform (^60^Co and ^133^Ba): 27 Not conform (^133^Ba only): 31 Not conform (^137^Cs only): 30 Not conform (^40^K only): 37, 14 No result: 16, 26, 40
13528	Acceptable (all RN): 2, 3, 4, 5, 6, 10, 11, 12, 14, 15, 17, 18, 20, 21, 22, 29, 35, 38, 41, 42, 43 Acceptable (^60^Co, ^133^Ba and ^137^Cs only): 1, 7, 9, 19, 23, 24, 25, 32, 33, 37 Acceptable (^133^Ba and ^137^Cs only): 13 Acceptable (^60^Co and ^40^K): 31 Acceptable (^60^Co only): 8 Acceptable (^133^Ba only): 30, 34, 36 Acceptable (^137^Cs only): 27 Action signal (^60^Co, ^133^Ba and ^137^Cs only): 28, 39 Action signal (^60^Co and ^133^Ba only): 27 Action signal (^133^Ba and ^137^Cs only): 31 Action signal (^137^Cs only): 30 Action signal (^40^K only): 37 Warning signal (^60^Co, ^137^Cs and ^40^K only): 36 Warning signal (^60^Co and ^137^Cs only): 34 Warning signal (^137^Cs only): 8 Warning signal (^60^Co only): 13 Warning signal (^40^K only): 28 No result: 16, 26, 40

**Table 4:** Compliance report for Task 3.1 and Task 3.2 “Medicine”: ^68^Ga/^68^Ge, ^88^Y and ^40^K

ISO standards	Task 3 “Medicine” #1 (^68^Ga, ^88^Y and ^40^K)	Task 3 “Medicine” #2 (^68^Ga, ^88^Y and ^40^K)
28218	Conform (all RN): 4, 22, 25, 32, 38, 41, 42, 43 Conform (^88^Y and ^40^K only): 5 Conform (^68^Ga and ^88^Y only): 8 Conform (^88^Y only): 1, 13, 34, 37 Not conform (^88^Y only): 30 Not conform (^40^K only): 1, 37	Conform (all RN): 2, 21, 29, 31, 33, 35 Conform (^88^Y and ^40^K only): 3, 6, 11, 12, 17, 18, 20, 36 Conform (^68^Ga and ^88^Y only): 9, 24 Conform (^88^Y only): 7, 14, 19, 27 Conform (^40^K only): 10 Not conform (^88^Y and ^40^K only): 28, 39 Not conform (^40^K only): 14
13528	Acceptable (all RN): 4, 22, 25, 38, 41, 42, 43 Acceptable (^68^Ga and ^88^Y only): 8, 32 Acceptable (^88^Y only): 1, 5, 13, 34, 37 Action signal (^88^Y only): 30 Action signal (^40^K only): 1, 37 Warning signal (^40^K only): 5, 32	Acceptable (all RN): 2, 21, 29, 33, 35 Acceptable (^68^Ga and ^88^Y only): 9, 24 Acceptable (^88^Y and ^40^K only): 3, 6, 11, 12, 14, 17, 18, 20, 31, 36 Acceptable (^88^Y only): 7, 19, 27 Acceptable (^40^K only): 10 Action signal (^88^Y and ^40^K only): 28 Action signal (^88^Y only): 39 Warning signal (^40^K only): 39 Warning signal (^68^Ga only): 31

**Table 5:** Compliance report for Task 4a “Calibration” (P4): ^133^Ba, ^152^Eu and ^40^K

ISO standards	Task 4a “Calibration” P4 (^133^Ba, ^152^Eu and ^40^K)
28218	Conform (all RN): 2, 3, 4, 5, 6, 12, 17, 21, 29, 32, 36, 41 Conform (^133^Ba only): 8, 30 Conform (^133^Ba and ^152^Eu only): 9, 14, 20, 24, 25, 33 Conform (^133^Ba and ^40^K only): 31 Not conform (^152^Eu and ^40^K only): 30 Not conform (^152^Eu only): 31 Not conform (^40^K only): 14, 20, 33
13528	Acceptable (all RN): 2, 3, 4, 5, 6, 12, 14, 17, 20, 21, 32, 33, 41 Acceptable (^133^Ba and ^152^Eu only): 9, 24, 25 Acceptable (^152^Eu and ^40^K only): 29 Acceptable (^133^Ba and ^40^K only): 30 Acceptable (^133^Ba only): 8 Acceptable (^40^K only): 31, 36 Action signal (^133^Ba and ^152^Eu only): 31 Warning signal (^133^Ba and ^152^Eu only): 36 Warning signal (^133^Ba only): 29 Warning signal (^152^Eu only): 30

**Table 6:** Compliance report for Task 4b “Calibration” (P5): ^133^Ba, ^152^Eu and ^40^K

ISO standards	Task 4b “Calibration” P5 (^133^Ba, ^152^Eu and ^40^K)
28218	Conform (all RN): 2, 3, 4, 5, 6, 10, 12, 17, 18, 20, 21, 29, 32, 36, 38, 41 Conform (^133^Ba and ^152^Eu only): 1, 7, 9, 13, 14, 16, 19, 24, 25, 31, 35 Conform (^152^Eu and ^40^K only): 33 Conform (^133^Ba only): 8, 30 Not conform (all RN): 39 Not conform (^133^Ba only): 33 Not conform (^152^Eu only): 30 Not conform (^40^K only): 1, 31 No result: 11, 15, 22, 23, 26, 27, 28, 34, 37, 40, 42, 43
13528	Acceptable (all RN): 2, 3, 4, 5, 6, 10, 12, 14, 17, 18, 20, 21, 29, 32, 36, 38, 41 Acceptable (^133^Ba and ^152^Eu only): 1, 7, 9, 13, 19, 24, 25, 35 Acceptable (^133^Ba only): 8, 16, 30 Acceptable (^40^K only): 33 Action signal (all RN): 39 Action signal (^133^Ba and ^152^Eu only): 31, 33 Action signal (^40^K): 1 Warning signal (^152^Eu only): 16, 30 Warning signal (^40^K only): 31 No result: 11, 15, 22, 23, 26, 27, 28, 34, 37, 40, 42, 43

## Footnotes

1 Q/Hampel method according to ISO 13528: https://quodata.de/en/web%C2%ADservices/QHampel.html#0
